# P-798. Non-Tuberculosis Mycobacterial Infections at a National Cancer Institute-Designated Comprehensive Cancer Center in Florida

**DOI:** 10.1093/ofid/ofae631.990

**Published:** 2025-01-29

**Authors:** Yanina Pasikhova, Matthew Snyder, Austin Morrison, Guy Handley, Misbahuddin Syed, Anthony P Cannella

**Affiliations:** Moffitt Cancer Center, Tampa, Florida; Moffitt Cancer Center, Tampa, Florida; Moffitt Cancer Center, Tampa, Florida; University of South Florida, Tampa, FL; Moffitt Cancer Center, Tampa, Florida; Moffitt Cancer Center and University of South Florida Morsani College of Medicine, Tampa, Florida

## Abstract

**Background:**

Non-tuberculous mycobacteria (NTM) are environmental organisms which become opportunistic pathogens in certain patient populations. NTM prevalence has increased globally, thought to be due to the increased diagnostic testing and availability of population-based studies. This is especially true in Florida, where we have previously documented more cases of *Mycobacterium abscessus* group compared to *M. avium* Complex (MAC) group. Current literature of NTM infections in oncology patients is minimal and represents a unique area for study. Here we present our epidemiological and clinical presentation of NTM infection cases at a National Cancer Institute-designated comprehensive cancer center.

**Figure 1.**
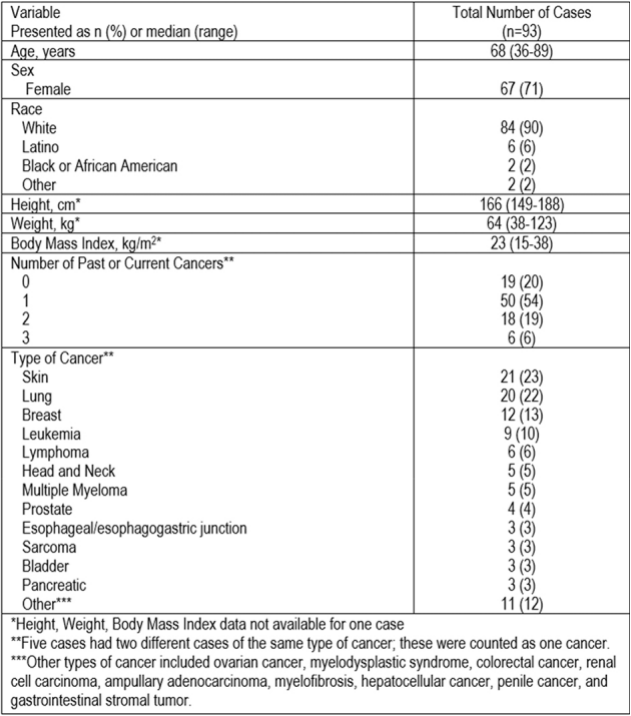
Patient Characteristics

**Methods:**

Retrospective, Institutional Review Board-approved, single-center cohort study, of adult patients with microbiologically proven NTM infections who presented from 2020 to 2023. Demographic, laboratory, and susceptibility data were retrieved from the electronic medical record system and evaluated to show institutional patterns.

**Figure 2.**
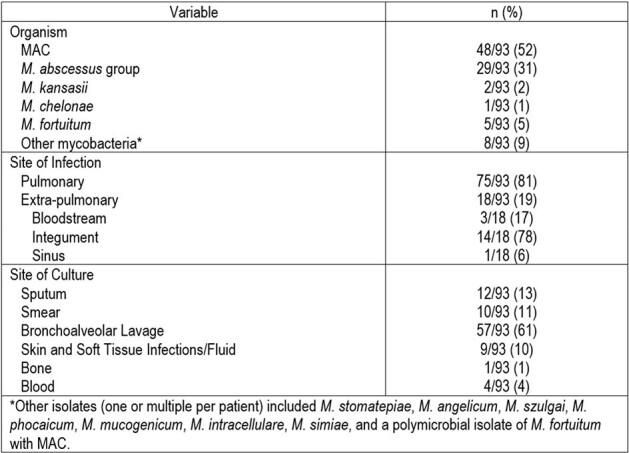
Microbiological Characteristics for Overall Population

**Results:**

Ninety-three cases of NTM infection met assessment criteria. Baseline characteristics are shown in Figure 1. A large proportion were female (71%) and white (90%). Many had a past or active cancer (80%), with skin (23%), lung (22%), and breast (13%) most prevalent. MAC (52%) and *M. abscessus* group (31%) were the most common NTM isolates (Figure 2). The majority were pulmonary infections (81%), with the integument accounting for most non-pulmonary sites (78%). Positive cultures were most frequently obtained via bronchoalveolar lavage (62%) followed by sputum (13%). Figure 3 offers insight into infection/culture site and cancer type stratified by organism, while susceptibility patterns are summarized in Figure 4.

**Figure 3.**
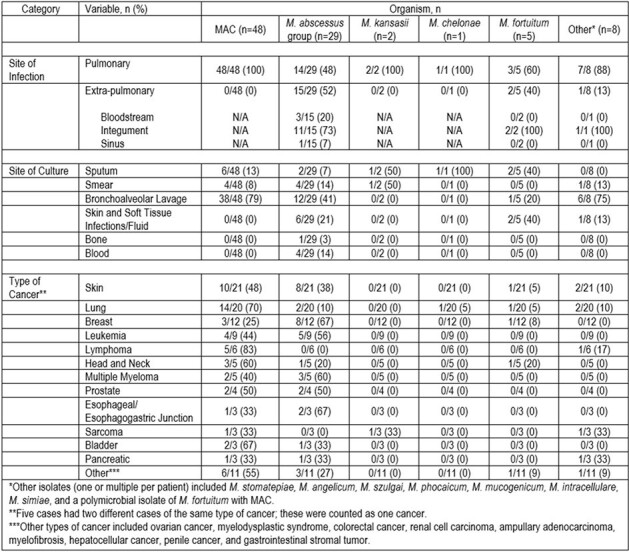
Site of Infection/Culture and Type of Cancer Stratified by Organism

**Conclusion:**

NTM infections at our institution were primarily caused by MAC or *M. abscessus* group and were typically pulmonary in nature, isolated via bronchoalveolar lavage. Most occurred in patients with a solid tumor malignancy. Further research is needed to delineate prevalence patterns, microbiological characteristics, and treatment approaches of NTM infections in our site’s population.

Susceptibility by Organism

**Figure:**
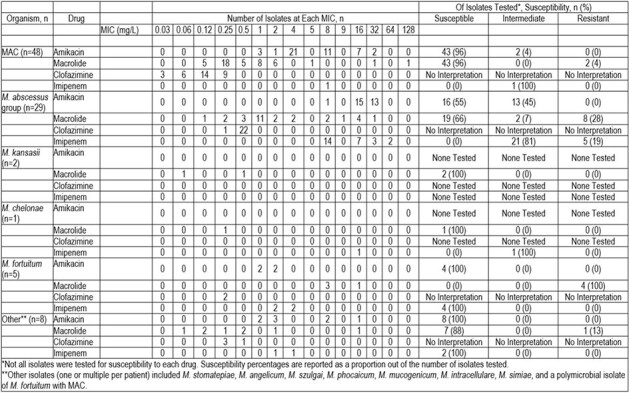

**Disclosures:**

**All Authors**: No reported disclosures

